# Seed-Encapsulation of Desiccation-Tolerant Microorganisms for the Protection of Maize from Drought: Phenotyping Effects of a New Dry Bioformulation

**DOI:** 10.3390/plants11081024

**Published:** 2022-04-09

**Authors:** Inês Rebelo Romão, Ana Sofia Rodrigues dos Santos, Leonardo Velasco, Elsa Martínez-Ferri, Juan Ignacio Vilchez, Maximino Manzanera

**Affiliations:** 1 Instituto de Tecnologia Química e Biológica (ITQB)—NOVA Lisboa, 2780-157 Oeiras, Portugal; ines.rromao@itqb.unl.pt (I.R.R.); anasofiasantos@itqb.unl.pt (A.S.R.d.S.); 2 Instituto Andaluz de Investigación y Formación en Agricultura, Pesca, Alimentación y Producción Ecológica (IFAPA Churriana), 29140 Málaga, Spain; leonardo.velasco@juntadeandalucia.es (L.V.); elsa.martinez@juntadeandalucia.es (E.M.-F.); 3 Institute for Water Research, Department of Microbiology, University of Granada, 18003 Granada, Spain

**Keywords:** desiccation-tolerant microorganisms, biofertilizer formulation, capsuled seeds, drought tolerance, crops enhancement, plant growth-promoting bacteria

## Abstract

Droughts and high temperatures deeply affect crop production. The use of desiccation-tolerant (or xerotolerant) microorganisms able to protect plants from droughts represents a promising alternative. These xerotolerant microorganisms have previously been used to modulate plant responses and improve their tolerance to drought. In addition, these microorganisms could be stored and used in dry formats, which would improve their viability and resilience at a much lower cost than current market alternatives. In the present study we analyze the possibility of using strains of xerotolerant Actinobacteria in encapsulated format on seeds. Under this formulation, we carried out greenhouse with farming soil with maize plants. Under greenhouse conditions, the plants showed greater resistance to drought, as well as increased growth and production yield, but not as well in field trials. This alternative could represent a useful tool to improve water efficiency in crops for drought-affected areas or affected by water scarcity.

## 1. Introduction

During the last decade, research of improvement of plant growth promotion, protection against pests or tolerance to various abiotic stresses based on microorganisms, has increased markedly [1]. This has led to an increase in biofertilizer products that has generated an increasingly important volume of business [2,3,4]. However, despite this, the number of candidate strains studied is much greater than the one that finally achieves commercial success or ends up being an applicable product. In most cases, the application of the strain with potential as a biofertilizer or biocontrol does not have the same success in the laboratory as in the open field [5,6]. Changing natural factors (humidity, soil type, pH, temperature, salinity…), competition with the local microfauna for nutrients or space, or predation on the applied inoculum, usually condition the success of the final product [7]. Although some strains are capable of performing under these conditions, most candidate strains need a very diverse formulation for their application to be successful.

The applicative formulations are very varied and can be adjusted according to the needs, including from dry powder o gel, liquid, or even mix products [8,9]. The application also can be divided in soil-applied and seed/root-coating alternatives. In this sense, the application on seeds has more options since it can be carried out from additive compounds to other fertilizers, soil structuring agents, granules, spray or irrigation [10]. However, the application on seed requires a more complex technical procedure. In most cases, the biofertilizer is applied as a bath to the seeds before sowing them, or by preparing an envelope or capsule over them [10,11]. The difference usually resides in the time that the treated product can be stored, since the wrapping or encapsulation process usually increases the product’s shelf-life. On the other hand, many strains are sensitive to capsule drying processes and lose viability or effectiveness. In this case, drought-tolerant biofertilizers acquire special relevance because such strains don’t require any adjuvant to guarantee their stability.

Drought-tolerance enhancing strains are those capable of inducing changes in the way plants cope with water deficit situations. In this sense, these strains can improve the structure of the soil to retain water (by production of organo-polysaccharides and proteins as golmalin, mucilages and hydrophobins), induce the formation of roots and hairy roots to access more sources of water (e.g., production of auxins), or regulate the response to drought stress (e.g., production of 1-aminocyclopropane-1-carboxylic acid deaminase (ACCd) to control ethylene levels during stress) [12,13,14]. Furthermore, xerotolerant strains are capable of producing and exuding exopolysaccharides, extracellular DNA (exDNA) and osmo- and xeroprotectant compounds that retain water in their structure and protect sensible structures. Thus, we have described strains of the *Microbacterium*, *Arthrobacter*, *Pseudoarthrobacter*, *Leucobacter* and *Rhodococcus* genera as capable of protecting pepper plants against drought using some of these mechanisms [15,16,17,18]. Moreover, some other strains of these genera were reported previously as drought stress tolerant [19,20,21].

This kind of strains are capable of overcoming many of the storage, manipulation and application difficulties, since they remain viable for longer in dry formulations. Likewise, in general, they tend to better resist changing environmental conditions and abiotic stresses. Furthermore, their preparation for application requires much less additives or adjuvants to ensure their viability. Consequently, these strains can represent an important improvement when applying as biofertilizer or stress-response enhancer product for plants, since large quantities of viable cells will not be required to ensure viability, reducing as well preparation, storage and distribution costs [22,23]. Hence, we propose the use of xerotolerant strains capable of promoting plant growth and improving drought tolerance in the plants, formulated in plastic capsules on the seeds. In this work we have tested the efficacy of this formulation over time, the evaluation resilience in soil, as well as the beneficial effects on plants.

## 2. Results

### 2.1. Strains Survival in Coating Formulation, Capsuled Seeds Germination and Resilience Evaluation in Soil

**Figure 1:**
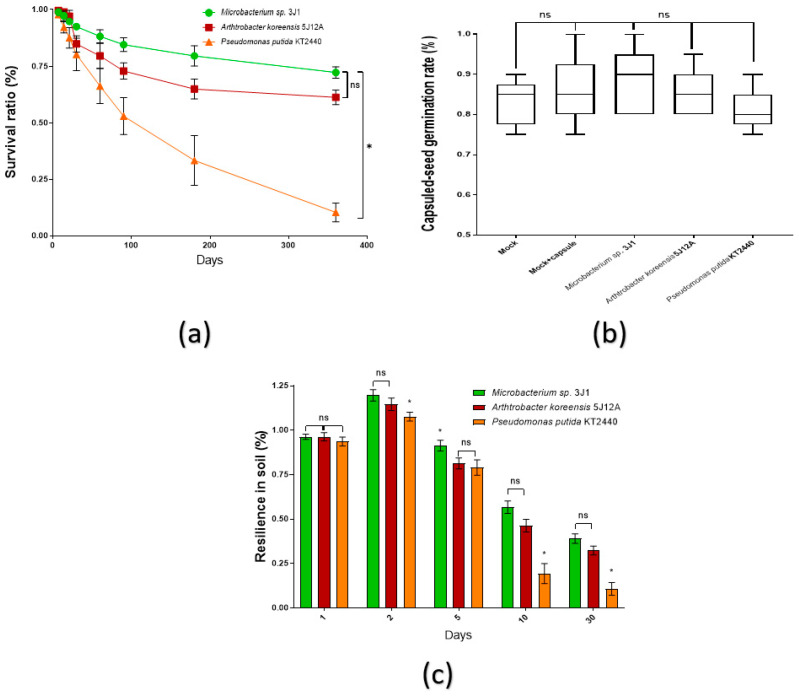
Quality control in dry capsule bioformulation. (**a**) The strains’ survival ratio in this formulation is showed along a year of room temperature stock. The line graph shows data from three independent experiments (*n* = 9 biologically independent samples); (**b**) percentage of maize seeds germination was recorded for a week to evaluate possible interference of the bioformulation. The boxplots show representative data from three independent experiments (*n* = 60). Whiskers represent the minimum to maximum data range, and the median is represented by the central horizontal line. The upper and lower limits of the box outline represent the first and third quartiles; (**c**) finally, during 30 days, percentage of strains’ resilience in soil was recorded. The bar graph shows data from three independent experiments (*n* = 9 biologically independent samples). In these panels, sets of data were compared by two-tailed Student’s *t*-test and 95% confidence intervals. Error bars represent s.d.; significance of statistical difference: *, significance by *p* < 0.05; ns, not significant.

As a quality control of the capsule bioformulation, the survival of strains along the time under capsule formulation was recorded as a way to ensure their stability over time during processing and storing. Thus, during the first month, survival rate of Arthrobacter koreensis 5J12A and Pseudomonas putida KT2440 was reduced in about 10%, however *Microbacterium* sp. 31J survival decreased to that level only by second month of room temperature storing. By the twelfth month, survival rate of *Microbacterium* sp. 31J, A. koreensis 5J12A and *P. putida* KT2440 dropped down to 72, 62 and 10%, respectively (Figure 1a). A single type of colony was found in each test showing the same appearance of the colonies of the used microorganisms. Therefore, contamination with other microorganisms was disregarded.

After assessing for how long are viable the strains in the capsule formulation, we tested their influence over germination rate once coating the maize seeds. Thus, germination rate in soil after a week was not affected by the treatments, keeping a consistent rate about 80–90% in all capsuled conditions (with or without strains), with no significant difference respect to regular uncoated seeds (Figure 1b). 

In order to determinate the resilience of bacteria in soil, their recovery was monitored during 30 days. Compared to original soil inoculation with capsules, after 2 days in soil, percentage of CFUs/g recovered ascended for *Microbacterium* sp. 31J, A. koreensis 5J12A and *P. putida* KT2440 up to 20, 25 and 8%, respectively. This is probably due to a progressive capsule releasing process that peaks at that time. After 5 days, recovery percentage still remained above 80% for all the strains. However, by day 10 recovery of *P. putida* KT2440 fell below 20%, still remaining above 50% for strains *Microbacterium* sp. 31J and A. koreensis 5J12A. By the end of the test, after 30 days, *Microbacterium* sp. 31J strain recovery was about 40% compared with 30% for *A. koreensis* 5J12A (Figure 1c). 

### 2.2. Laboratory Tests

As a previous step to the tests with natural soil in the greenhouse, studies under a controlled environment in a laboratory growth chamber were carried out. In these experiments, the efficiency and final effect of the candidate bacterial strains were evaluated when they were dispensed in liquid and capsule formats. The differences obtained in both tests suggest that the treatment with capsules is more efficient in terms of the number of bacteria required, probably due to the greater dispersion of the liquid formulation in the soil. 

**Figure 2:**
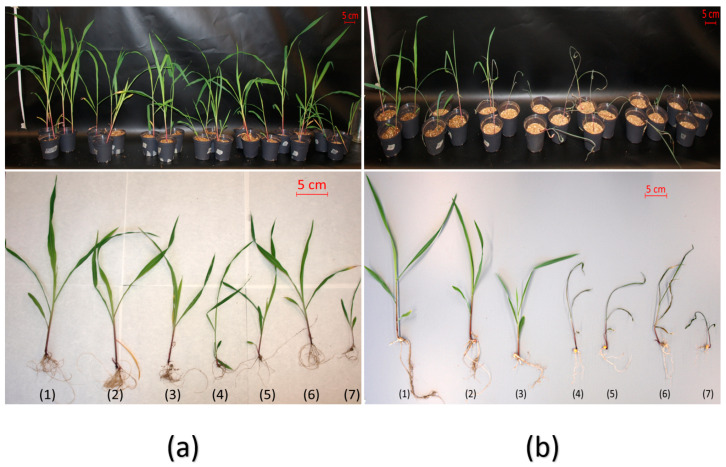
Phenotype registered in maize plants under capsuled-seed treatment in lab tests. (**a**,**b**) These pictures show the 14 DAT phenotype of seedlings treated with capsuled xerotolerant strains under regular irrigation (**a**) and drought (**b**) conditions, both in pots and in full-plant view. Applied treatments were: (1) *Microbacterium* sp. 31J, (2) *Arthrobacter koreensis* 5J12A, (3) *Rhodococcus globerulus* 4J2A2, (4) *Leucobacter* sp. 4J7B1, (5) *Pseudarthrobacter siccitolerans* 4J27, (6) *Pseudomonas putida* KT2440 and (7) mock. Three repetitions of three replica (*n* = 9) were prepared for this test. Red bar indicates a 5-cm reference in the picture.

Thus, the results obtained in laboratory conditions with capsule formulation of *Microbacterium* sp. 31J and A. koreensis 5J12A were similar to those previously recorded for other crops using liquid format. Moreover, no phenotypic variations were recorded by using capsules as carrier for those other xerotolerant strains Rhodococcus globerulus 4J2A2, *Leucobacter* sp. 4J7B1 and Pseudarthrobacter siccitolerans 4J27 as inoculant, under both stressing and regular conditions (Figure 2a,b). Consequently, in the following tests only strains *Microbacterium* sp. 31J and *A. koreensis* 5J12A were evaluated as reference for effects on plants.

**Figure 3:**
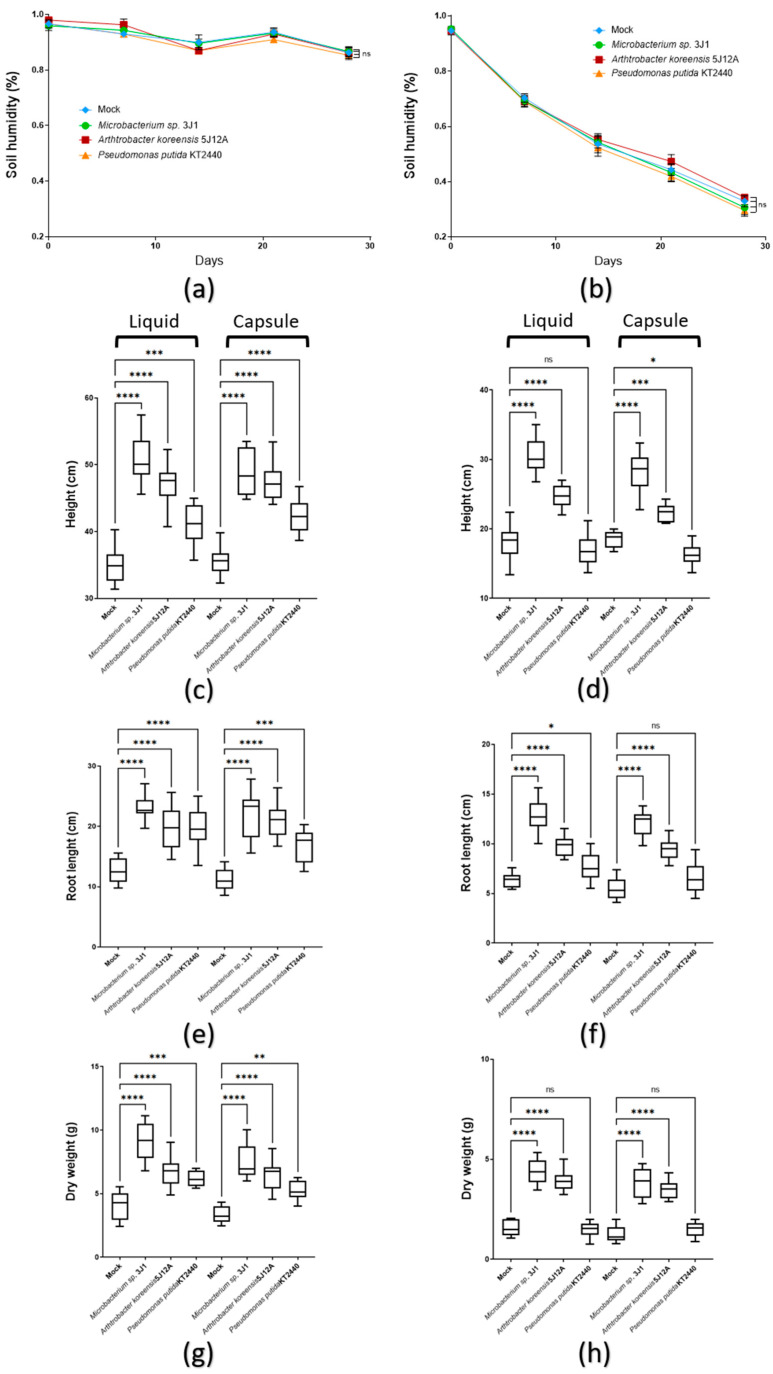
Capsule vs. liquid treatment effects on 30 DAT maize plants. (**a**,**b**) The line graphs show weekly soil humidity representative data from three independent experiments (*n* = 9) under regular irrigation (**a**) and drought conditions (**b**); (**c**,**d**) boxplot graphs record the height achieved by plants differentially inoculated with liquid and capsule formats, under regular irrigation (**c**) and drought conditions (**d**); (**e**,**f**) boxplot graphs record the root length in plants differentially inoculated with liquid and capsule formats, under regular irrigation (**e**) and drought conditions (**f**); (**g**,**h**) boxplot graphs record the dry weight biomass of plants differentially inoculated with liquid and capsule formats, under regular irrigation (**g**) and drought conditions (**h**). All the boxplots in these panels show representative data from three independent experiments (*n* = 60). Whiskers represent the minimum to maximum data range, and the median is represented by the central horizontal line. The upper and lower limits of the box outline represent the first and third quartiles. Error bars represent s.d.; Significance of statistical difference: *, significance by *p* < 0.05; **, significance by *p* < 0.01; ***, significance by *p* < 0.001; ****, significance by *p* < 0.0001; ns, not significant.

To control soil conditions, humidity was recorded to control water effects on each treatment. For regular irrigation tests, humidity was consistently kept about 85% during all test; under drought conditions, values were similarly declining in all treatments till about 30% (Figure 3a,b).

In regular irrigation tests with liquid inoculum, compared to non-inoculated control plants (mock), the strain that caused the greatest growth promotion in terms of height, root length and total dry biomass produced on maize plants was *Microbacterium* sp. 31J (25%, 20%, 33%, respectively), followed by *A. koreensis* 5J12A (22%, 18%, 20%, respectively), which slightly exceeded those obtained by the control strain *P. putida* KT2440 (20%, 16%, 19%, respectively). In the case of the capsule formulation, these percentages were slightly increased only in the height and the total dry biomass in the treatment with *Microbacterium* sp. 3J1 (28%, and 35%, respectively) with respect to the treatments with liquid formulation. No significant differences were found under the other two treatments (Figure 3c,e,g).

Regarding the treatments under drought conditions, these showed greater efficiency with the use of formulation in capsules. In this sense, with respect to the non-inoculated control plants (mock), the plants inoculated with the capsule formulation showed a better response to stress than those that were inoculated with the liquid formulation. Thus, treatment with *Microbacterium* sp. 3J1 in capsule format showed values of height, root length and total dry biomass produced 5%, 3% and 3%, respectively, higher than those shown when the plants were inoculated with liquid formulation. Similarly, it was recorded in the plants inoculated with A. koreensis 5J12A (4%, 3% and 3% higher, respectively), not finding significant differences by formulation when the plants were inoculated with *P. putida* KT2440 (Figure 3d,f,h).

### 2.3. Greenhouse Tests

**Figure 4:**
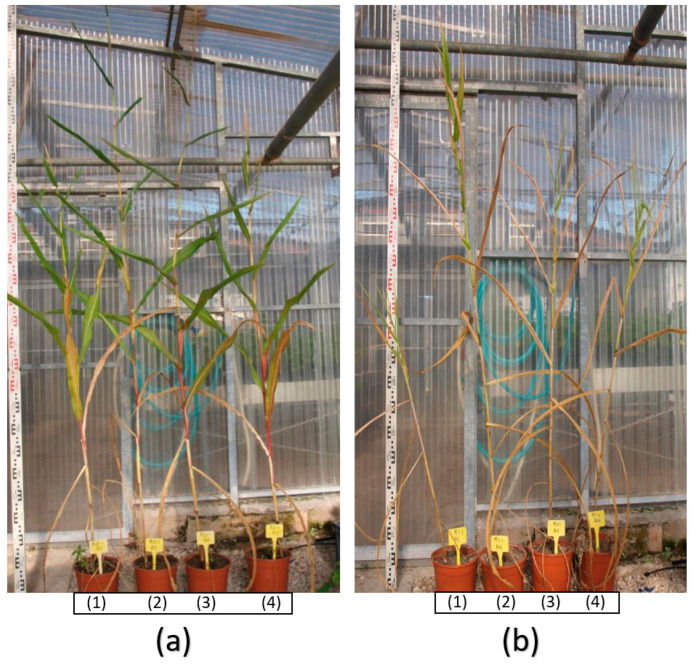
Phenotype registered in maize plants under capsuled-seed treatment in greenhouse tests. (**a**,**b**) These pictures show the 120 DAT phenotype of seedlings treated with capsuled xerotolerant strains under regular (**a**) and drought (**b**) conditions, both in pots and in full-plant view. Applied treatments were: (1) mock, (2) *Microbacterium* sp. 31J, (3) *A. koreensis* 5J12A and (4) *P. putida* KT2440. Three repetitions od three replica (*n* = 9) were prepared for this test. In the scale bars, each section corresponds to a 10-cm reference.

Given the above results, only the capsule formulation was selected for the greenhouse tests. The growth promotion (without water restriction) and drought tolerance tests were carried out in parallel under the same conditions. Strains inoculated with *Microbacterium* sp. 3J1 and *A. koreensis* 5J12A showed the better drought-resistance phenotypes at the end of the treatment (Figure 4a,b).

#### 2.3.1. Plant Growth Promotion

**Figure 5:**
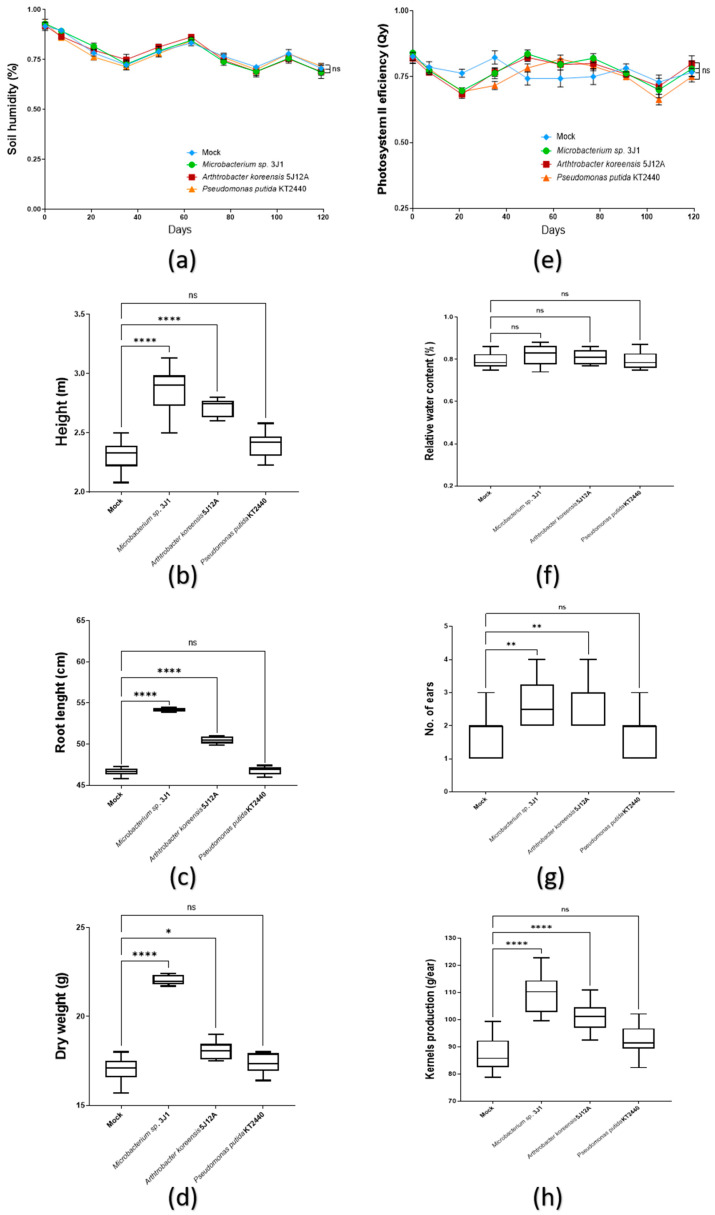
Evaluation of capsule treatment 120 DAT in maize plants under regular irrigation. (**a**) The line graph shows soil humidity representative data from three independent experiments (*n* = 9); (**b**) boxplot graphs record the height; (**c**) the root length; (**d**) and the dry weight biomass of the plants under testing. (**e**) The line graph shows the efficiency of photosystem II (Qy) along the test, as representative data from three independent experiments (*n* = 9); (**f**) boxplot graphs record the relative water content (RWC); (**g**) the number of maize ears; (**h**) and the kernels production in the plants under testing. All the boxplots in these panels show representative data from three independent experiments (*n* = 60). Whiskers represent the minimum to maximum data range, and the median is represented by the central horizontal line. The upper and lower limits of the box outline represent the first and third quartiles. Error bars represent s.d.; Significance of statistical difference: *, significance by *p* < 0.05; **, significance by *p* < 0.01; ****, significance by *p* < 0.0001; ns, not significant.

Soil humidity value of the pots was kept constant throughout the process, with a maximum variation of about 5% between pots (Figure 5a). Height and root length of the plants was studied together with the total dry biomass produced in order to evaluate the beneficial effects on the plant development. Thus, plants treated with *Microbacterium* sp. 3J1 (2.86 m ± 0.18) and *A. koreensis* 5J12A (2.71 m ± 0.07) showed a higher phenotype than those untreated plants (2.3 m ± 0.12), by 19% and 13%, respectively. Plants treated with *P. putida* KT2440 also shower higher phenotype (2.4 m ± 0.10), but this value was not significative enough (Figure 5b). This same situation was recorded in root length, arising 16% and 7% of increase when plants were treated with *Microbacterium* sp. 3J1 (54.5 cm ± 0.63) and *A. koreensis* 5J12A (50.5 cm ± 0.38) (Figure 5c). However, an increase of biomass under these conditions was only significative under the *Microbacterium* sp. 3J1 treatment (22 g ± 0.26), recording up to a 30% more dry biomass than in mock condition (17 g ± 0.65) (Figure 5d). 

On the other hand, the efficiency values of the photosystem II efficiency (Qy) registered, showed a minimum variation during the test, always being between 0.74 and 0.85 in all the treatments. There were no significant differences between the treatments during the trial (Figure 5e). Regarding the WRC values, which indicate the water state of the plant with respect to the best possible, in general similar record was observed, with values oscillating form 0.75 up to 0.88 under all treatments by the end of the test (Figure 5f). No statistical differences were recorded between each treatment, meaning their water status was similar during the test in all of them.

Analyzing yield achieved under different treatments, number of ears produced and weight of kernels were recorded. Ears produced by plants treated with *Microbacterium* sp. 3J1 (2.7) and *A. koreensis* 5J12A (2.8 g) were about 65% higher when compared with those produced by untreated plants and plants treated with *P. putida* KT2440, that produced an average of 1.7 ears per plant (Figure 5g). The weight of kernels produced by plants treated with *Microbacterium* sp. 3J1 (109.5 g ± 7.07) and *A. koreensis* 5J12A (101.2 g ± 5.39) was 25 and 16% higher respectively, than by untreated plants. Those inoculated with *P. putida* KT2440 didn’t show statistical difference respect to mock conditions (Figure 5h).

#### 2.3.2. Drought Tolerance Test

**Figure 6:**
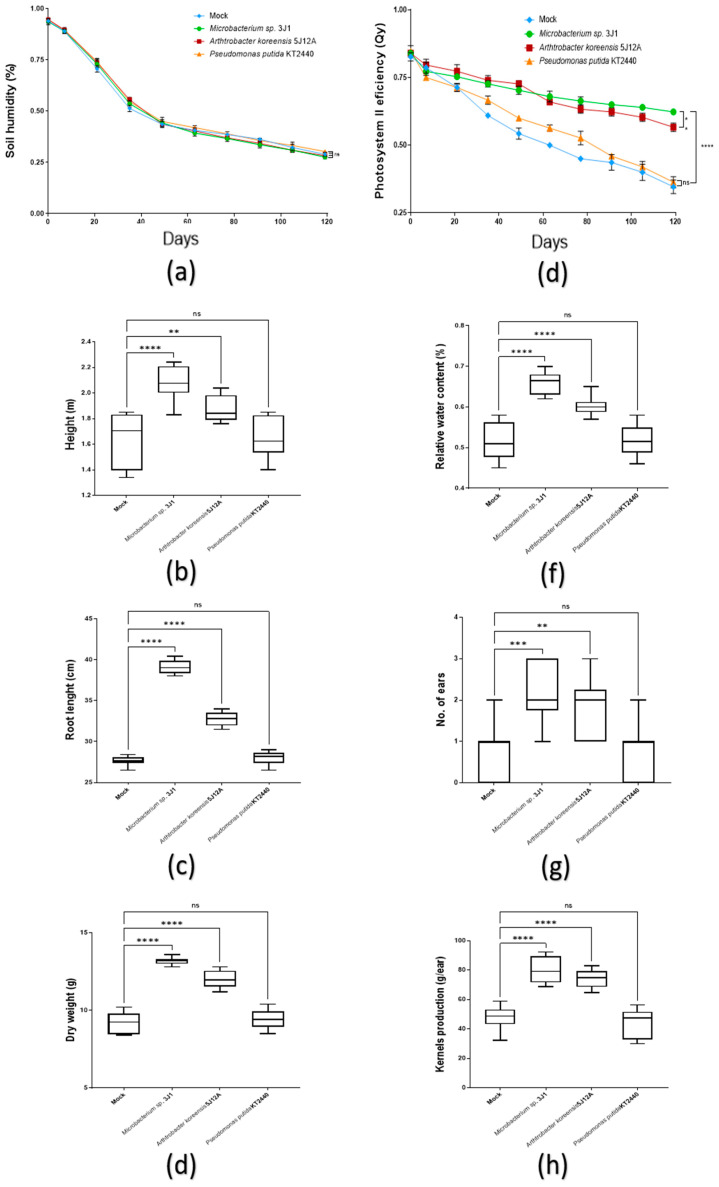
Evaluation of capsule treatment 120 DAT in maize plants under drought conditions. (**a**) The line graph shows soil humidity representative data from three independent experiments (*n* = 9); (**b**) boxplot graphs record the height; (**c**) the root length; (**d**) and the dry weight biomass of the plants under testing. (**e**) The line graph shows the efficiency of photosystem II (Qy) along the test, as representative data from three independent experiments (*n* = 9); (**f**) boxplot graphs record the relative water content (RWC); (**g**) the number of maize ears; (**h**) and the kernels production in the plants under testing. All the boxplots in these panels show representative data from three independent experiments (*n* = 60). Whiskers represent the minimum to maximum data range, and the median is represented by the central horizontal line. The upper and lower limits of the box outline represent the first and third quartiles. Error bars represent s.d.; Significance of statistical difference: *, significance by *p <* 0.05; **, significance by *p* < 0.01; ***, significance by *p* < 0.001; ****, significance by *p* < 0.0001; ns, not significant.

Considering the above data as a development reference under non-stressful conditions, we evaluated the effects of inoculation treatments with capsules under drought conditions. Soil humidity value of the pots gradually decreased at a rate of 3–5% per week, except in the first and last week, where the decreased in humidity reached 7%. The minimum value recorded was around 30% by the end of the test (Figure 6a). In total, registered decrease was about 60% during the process. There was no significant difference in moisture loss between the tested conditions. At least 20 plants were monitored per treatment to evaluate their development by height, root length and total dry biomass produced. On the other hand, we also recorded the efficiency of the photosystem II efficiency (Qy) and relative water content as evaluation of their stress response during the process. Under these conditions, plant treated with *Microbacterium* sp. 3J1 and *A. koreensis* 5J12A showed an enhanced drought-tolerance phenotype respect to those treated with *P. putida* KT2440 and to untreated plants.

Thus, height recorded by the end of the test by the untreated plants (1.62 m ± 0.20), as well as by the plants inoculated with *P. putida* KT2440 (1.65 m ± 0.15), showing no significant difference between both treatments. Reversely, plants treated with *Microbacterium* sp. 3J1 (2.08 m ± 0.12) or *A. koreensis* 5J12A (1.90 m ± 0.1) showed values up to 25% and 15%, respectively, higher than control (Figure 6b). On the other hand, length of the root at the end of the test in plants treated with *P. putida* KT2440 and in those untreated was about 28 cm, showing no significant difference between both conditions. For those plants treated with *Microbacterium* sp. 3J1 (39.1 cm ± 0.75) and *A. koreensis* 5J12A (32.8 cm ± 0.8), root length shown about 30% and 15% increase compared to that shown by the untreated plants (Figure 6c). With respect to the total dry biomass produced, untreated plants (9.2 g ± 0.67), and those treated with *P. putida* KT2440 (9.4 g ± 0.55), again showed not significative difference. On the contrary, plants treated with *Microbacterium* sp. 3J1 (13.2 g ± 0.22) or *A. koreensis* 5J12A (12 g ± 0.55) shown a significant increase about 45% and 30%, respectively, compared to that shown by the mock condition plants (Figure 6d).

Comparing treatments in terms of drought response, photosystem II efficiency (Qy) registered in plants treated with *P. putida* KT2440 and untreated plants showed lower values than 0.6 during the second month of treatment, finishing the test with about 0.35–0.4, indicating a state of wilting. Plants treated with *Microbacterium* sp. 3J1 or *A. koreensis* 5J12A revealed as well a slow decrease in values along the test, but they were never below 0.6, showing a better tolerance level (Figure 6e). Regarding the RWC values, in general they showed a remarkable drop (about 0.5) in plants treated with *P. putida* KT2440 and in those untreated by the end of the test, showing no significant difference between both conditions. However, WRC of the plants treated with *Microbacterium* sp. 3J1 (0.67) or *A. koreensis* 5J12A (0.63) showed significative differences respect to mock condition plants (Figure 6f).

With respec to yield parameters, number of ears produced by plants treated with *Microbacterium* sp. 3J1 (2.1) was about 29% lower than in regular irrigation conditions, but producing 3 times more than untreated plants under drought conditions. In the case of plants treated with *A. koreensis* 5J12A (1.8), number of ears produced was 65% lower under drought conditions, similarly to those produced by untreated plants under regular irrigation conditions. On the other hand, plants treated with *P. putida* KT2440 (0.8) produced half than in regular irrigation conditions (as in case of untreated plants) (Figure 6h). With respect to the weight of kernels produced per ear, plants treated with *Microbacterium* sp. 3J1 (80.1 g ± 8.20) and *A. koreensis* 5J12A (74.2 g ± 5.91) registered about 70% less weight, but still about 70 and 55%, respectively, more than untreated plants. Those inoculated with *P. putida* KT2440 produced half than in regular irrigation conditions, but didn’t show statistical difference respect to mock conditions (Figure 6h).

## 3. Discussion

**Figure 7:**
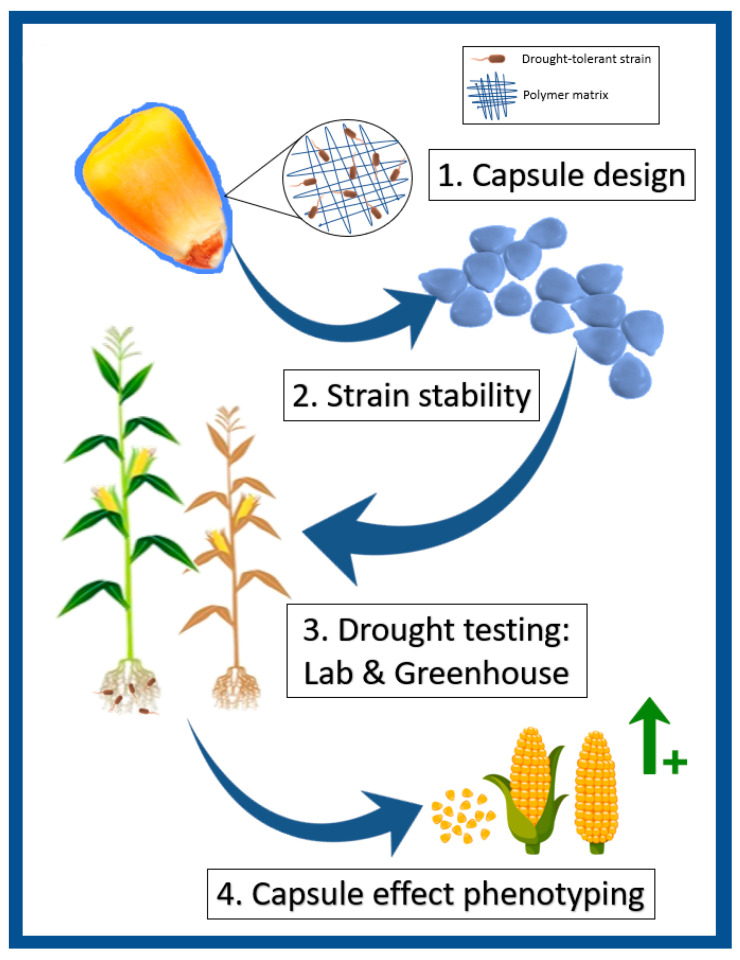
Evaluation workflow for new capsules bioformulation in maize. Schematic description of the preparing, application and evaluation process for dry capsule bioformulation with xerotolerant strains.

Our work seeks to evaluate the use of xerotolerant organisms with the capacity to promote growth as drought tolerance inducers in plants, using a dry formulation to encapsulate seeds. For this, we applied a quality control system on the new formulation, ensuring the presence, viability and resilience of the strains, as well as the germination of the seeds encapsulated with this formulation. In this study we used maize as a model plant, carrying out consecutive laboratory and greenhouse tests to ensure the effects of this new formulation. In addition, we evaluated the general productivity patterns in plants under treatment, in both regular irrigation and drought stress conditions, obtaining a comprehensive and coherent model (Figure 7).

Hence, the final aim in this work was to carry out an evaluation in the use of inert materials as carriers for dry-formulated biofertilizers. Our candidate bacteria are characterized as xerotolerant, which allow us to design a full-dry formulation, which make the stability of the system not water- or temperature-dependent. This implicates that characteristic of tentative carrier must ensure the complete absence of water in their structure, but at the same time guarantee a matrix that stabilizes the bacteria included in the formulation. In this sense, we decided to use a polymeric matrix, achieved by the dissolution of expanded polystyrene or porexpan (EPS). This material causes a huge amount of waste annually, resulting our formulation in a way to revalue discarded and polluting materials [24,25]. Although the dilute configuration of this polymer is easier to degrade in the medium, we are aware that the dispersion of this compound in a generalized way could produce associated contamination (microplastics). For this, we only consider this approach as a first contact towards a more suitable material. Finally, the carrier agent must be able to be applied in a simple and economically viable way [26]. Considering current seed-coating technology, our system can be perceived as cost-efficient as well.

Then, we aim to confirm the type of inert compounds and matrices necessary to maintain stable capsules and coatings under drought conditions, guaranteeing longer resilience times for biofertilizer formulations. Therefore, this type of capsule will allow its incorporation into seeds as a coating or even as a controlled dispersion granule, to guarantee the presence of viable cells for a longer time. Hence, our results indicate that the survival of strains over time in these capsules is more stable than previous models, without compromising the germination capacity of the seeds in the process. In addition, the resilience of the strains registered in soil is higher than that provided by other kind of carriers [8,26,27,28]. This causes that, despite carrying a proportionally lower number of cells, the polymeric matrix carrier indicates an efficiency similar to that shown by a liquid carrier, with a major initial number of viable cells but also bigger associated dispersion problems. In the future, this type of polymeric matrices should be made up of biodegradable binder structures and nanomaterials that also allow a more efficient controlled dispersion and the possible addition of other adjuvant substances to improve the effects of biofertilizers [27,28].

Attending again to the results obtained, after ensuring the formulation model with parallel laboratory tests, capsuled formulation was tested under greenhouse conditions, with natural soil as a more realistic validation. Although encapsulated seeds guarantee greater contact with the plants, and a longer resilience in the soil [27,29,30,31], plant physiological results in greenhouse were significantly lower than expected. This is probably due to problems in adaptation to the local flora and environment. Both in greenhouse and field conditions, difficulties have been described when applying bacterial strains as inoculants [32,33,34]. The effect of biological agents in the environment is one of the most described factors in the loss of efficiency for this kind of tests [35,36,37]. Competition, predation and displacement events by local soil organisms are commonly reported to not allow the establishment and distribution of the inoculated strain in the population [38,39]. In this way, protozoa, amoebae and nematodes, among others, may prey on populations of bacteria. Antimicrobial-producing strains can also control population changes between microorganisms [40] and the whole local microfauna is going to compete for nutritional and spatial resources more effectively as they are better adapted to the environmental conditions [41]. Finally, the dispersion by leaching caused by the irrigation is as well capable of diluting the inoculums, preventing a minimum population number that guarantees good contact and colonization with the plant [8,27,31]. This represents a very important impact factor on the interaction, especially when endophytic or persistent biofilm-forming strains are used [37]. In future, additional layers, supplementation with adjuvants or increasing the microbial load in the system could help to solve these problems and to improve the formulation efficiency.

Despite this situation, we report that the use of *Microbacterium* sp. 3J1 and *A. koreensis* 5J12A still improved overall plant growth (height, roots length, and dry biomass) under regular watering conditions in the greenhouse tests. In the case of *P. putida* KT2440 capsules, these did not show significant differences with the untreated plants. This condition means that the efficiency of this formulation resides both in the structure and the ability of the strains to survive the process without external stabilizing substances. Furthermore, under drought conditions, all the parameters recorded were reduced, but the more significant drop occurred in the amount of biomass produced when the capsules containing *Microbacterium* sp. 3J1 or *A. koreensis* 5J12A were used [16,17]. In this sense, it is important to notice that the evaluation of the efficiency of photosystem II (Qy) and the relative water content (RWC) throughout the process, as well as the general appearance of the plants, indicate that they are in better hydric state throughout the stressing process thanks to the effect of the applied bacteria. Finally, the yield registered from the plants under different treatments shows that, under normal irrigation conditions, again the plants inoculated with *P. putida* KT2440 were not able to produce more ears or increase the weight of the kernels when compared to untreated plants. On the other hand, plants treated with *Microbacterium* sp. 3J1 or *A. koreensis* 5J12A, enhanced their yield, even under drought conditions. Focusing on the plants treated with *Microbacterium* sp. 3J1, they practically maintained the total weight of grains produced per ear under drought conditions. These results are consistent and we consider them as very promising, however once the tests were performed in a field scale, their significance dropped down for plant growth-promotion effects, and were not significative enough in terms of the protection against drought [42] (see Annex 1). 

In light of these results, we consider that our carrier design (matrix system) and the natural xerotolerance achieved a controlled dispersion, increased soil resilience and enhanced inoculum performance under more realistic conditions. Nevertheless, the model still requires adjustments to cope with previously mentioned problems of adaptability to environmental conditions, particularly, for field use. Despite this, the huge potential of this xerotolerant strain-based, dry bioformulations, opens a frame of work fascinating for the future of the industry. The proposed formulation can be considered as innovative approach for this kind of biotechnology, as well as a way to enhance the application of biofertilizers in stressful environments. 

## 4. Materials and Methods

### 4.1. Culture and Preparation of the Strains

The strains *Microbacterium* sp. 3J1 (CECT7624) and *Arthrobacterium koreensis* 5J12A (CECT7626) [43,44], described as xerotolerant strains with the capacity to promote growth and protect against drought in previous studies [16,43,44], were used in this study. Complementarily, for the lab testing of capsules, we added other xerotolerant closely related strains with diverse drought-protection and plant-growth promotion skills as controls: *Rhodococcus globerulus* 4J2A2, *Leucobacter* sp. 4J7B1 and *Pseudarthrobacter siccitolerans* 4J27 [45,46,47]. Additionally, as a plant growth promoter control strain, *Pseudomonas putida* KT2440 was included. Bacterial strains were grown overnight in TSB (San Luis, MO, USA, Sigma-Aldrich) at 150 rpm and 30 °C until reaching OD_600nm_ = 1.0 (equivalent to 10^8^ CFU/mL) to use as inoculum. The grown cultures were centrifuged at 8000 rpm for 20 min and then resuspended in the same volume of M9 minimal medium (48 mM Na_2_HPO_4_, 22 mM KH_2_PO_4_, 9 mM NaCl, 19 mM NH_4_Cl, 0.1 mM CaCl_2_ and 2 mM MgSO_4_; supplemented with 5 mM glucose and micronutrients).

### 4.2. Preparation of Liquid Inoculum and Capsules. Assessment of the Viability of the Cells in Coating and Germination of Seeds Evaluation of Resilience in Soil Evaluation 

For liquid inoculum, M9 minimal medium was employed as carrier. On the other hand, for capsule formulation, expanded polystyrene (EPS) was diluted with chloroform as previously described by Manzanera and collaborators [30,48], and then mixed with bacteria lyophilized in trehalose 10% (*w*/*v*), as preservative preparing. Viable bacteria solutions adjusted up to 10^9^ CFU/mL were prepared for each formulation. In the case of capsules, strains to prevent their damage during the process. Bacteria-free liquid or capsule were used as control (mock). Fifty milliliters of liquid formulation were applied per pot as a bacterial treatment, meanwhile capsules were applied as a bath to cover maize seeds for 10 min with slight agitation till the formulation completely coated (around 1 mm thick). The seeds were stored at room temperature (25–30 °C) until the moment of use. Serial dilutions of the coating were prepared on the seeds to ensure a minimum of 10^8^ CFU/g of seed. 

Survival rate (%) of the strains in capsules was assessed by culturing in TSB medium and number of viable cells (NVCs) counting at 2, 7, 14, 21, 30, 90, 180 and 360 days of storage. In order to evaluate interference of coating with seeds development, germination rate of coated seeds was compared to uncoated seeds. For this purpose, we sowed 3 sets of 20 seeds in soil per treatment, and germination rate (%) was calculated after a week. Resilience of encapsulated strains was assessed as the survival of the bacterial cells in the substrate. Survival of the cell was assessed at 1, 2, 5, 10 and 30 days after capsule preparation. Number of viable cells at different times were compared with those corresponding to time 0. We used the recovery rate in 1 g of soil just after inoculation as the amount of initial CFUs, which served as a reference to express resilience as % recovery over time. To avoid interferences from other strains, sterile vermiculite was used as a substrate.

### 4.3. Laboratory Tests 

Maize seeds (*Zea mays* subsp. *mays*) were surface-sterilized for 15 min (15% commercial bleach + 0.01% Triton X-100) and washed three times with sterile double distilled water (H_2_O dd). In laboratory tests, 0.5 L pots were filled with 0.4 L of a mixture of vegetable substrate: vermiculite, in a 3:1 ratio (*v*:*v*). For liquid inoculation of strains, seeds were sown in the pots and treated with 40 mL of the liquid inoculant 1 day after germination. On the other hand, when strains were carried in capsule format on seeds, they were sown directly in the soil without further treatment. The treatments were considered at time 0 days-after-treatment (DAT) once they germinated and their stem reached 5 cm in height. For growth promotion tests, soil moisture was kept at 70% of humidity by watering every 2–3 days. However, for drought treatments, the irrigation was stopped once the stems of the plants reached 15 cm high, maintaining humidity not below 30%. Samples were collected 30 DAT and each sample consisted of at least 6 replicas.

### 4.4. Greenhouse Tests

In greenhouse tests, 5 L pots were filled with farmland soil collected from Andalusian Institute for Research and Training in Agriculture, Fisheries, Food and Ecological Production (IFAPA Churriana) (Málaga, Spain). These studies were carried out between April and July in 2012. For liquid inoculation, the seeds were sown in the pots. and they were treated with 125 mL of the liquid inoculant once they germinated. In the case of the inoculant as coating, the prepared seeds were sown directly in the soil without further treatment. The treatments were considered at time 0 once they germinated and their stem reached 2 cm in height. For the growth promotion treatments, the irrigations kept the soil humidity at 70% by an automatic drop-by-drop irrigation system every 2–3 days. For the drought treatments, the watering stopped once the plants reached 40 cm in height and avoiding that the humidity was less than 30%. At least 20 plant per treatment were monitored regularly till 120 DAT, when the experiment was ended.

### 4.5. Phenotype Evaluation

Plant growth promotion and drought tolerance enhancement were evaluated by the record of plant aerial part (height), root length, total dry biomass produced (DW) at the end of each experiment. Additionally, fresh (FW) and fully turgid weight (FTW) were recorded as well to calculate relative water content of the plant (RWC) (FW − DW)/(FTW − DW) × 100 [49]. As main stress monitoring parameter, efficiency of photosystem II (Qy) was recorded during the process by using FluorPen FP100 (Photon Systems Instruments, Drasov, Czech Republic). Soil moisture was recorded in both conditions to assess the water treatments with the HH2 Moisture Meter and ML3 ThetaProbe Soil Moisture Sensor (Delta-T Devices, Delta-T Devices, Cambridge, UK) to assess the water conditions were stable.

### 4.6. Statistical Study

Three independent tests were prepared in each procedure, and their statistical significances were assessed by two-tailed Student’s t-test and 95% confidence intervals, and when required, by one-way ANOVA with post-hoc Tukey. These analyses were performed with Prism v.9.0.0 software (GraphPad, San Diego, CA, USA).

## 5. Conclusions

From this study we can conclude that desiccation tolerance of the isolate determined the viability of the dry formulation of specific PGPRs, which was correlated with the resilience of the strains in soil under laboratory conditions. By encapsulating the drought-protecting microbial biofertilizers (*Microbacterium* sp. 3J1 and *A. koreensis* 5J12A) an increased effect of the protection of plants from water stress was observed with higher values of plant height, root length and total dry biomass of 3% or over when tested in laboratory conditions. In this respect we can conclude that the use of a dry-matrix system in the formulation enhanced the resilience of the strains in soil as well as their survival along the time, without conditioning seed germination process. Compared to a classical liquid formulation, the xeroprotection of maize with drought-protecting strains was enhanced by the use of dry, capsuled formulation, recommending this dry-matrix formulations over conventional liquid-based for the use of drought-protecting microbial biofertilizers.

Greenhouse experiments resulted in a growth promoting effect of the plants inoculated with *Microbacterium* sp. 3J1 under normal irrigation with significant differences of dry biomass when compared with the rest of conditions. However, the effect of both drought-protecting strains was even higher under water stress, showing significant differences for plants height, root length, dry biomass, and photosystem II efficiency (Qy) when compared with non-inoculated plants or with plants inoculated with *P. putida* KT2440. The improvement was extended to yield under water stress when maize plants were inoculated with the drought-protecting strains, resulting in higher number of ears produced and heavier kernels per ear. In consequence, we can claim that growth and yield results obtained under greenhouse conditions showed to improve the viability and yield production of corn when inoculated with the drought-protecting strains *Microbacterium* sp. 3J1 and *A. koreensis* 5J12A under water stress.

## Data Availability

Not applicable.
